# Choroidal mass secondary to mucinous cystadenocarcinoma of ovary in a young woman: a life-threatening metastasis

**DOI:** 10.22336/rjo.2021.39

**Published:** 2021

**Authors:** Saidatulakma Shariff, Khairy Shamel Sonny Teo, Wan Hazabbah Wan Hitam

**Affiliations:** * Department of Ophthalmology and Visual Sciences, School of Medical Sciences, Health Campus, Universiti Sains Malaysia; Hospital Universiti Sains Malaysia, Malaysia

**Keywords:** choroidal mass, ovary carcinoma, metastases

## Abstract

**Objective:** To report a case of choroidal mass secondary to mucinous cystadenocarcinoma of ovary in a young woman.

**Method:** A case report.

**Result:** A 21-year-old woman presented with insidious painless, progressive, central scotoma of the right eye for 5 weeks. She was disease free for 9 years after she underwent right salpingo-oophorectomy for her mucinous cystadenocarcinoma of right ovary. She completed 6 cycles of chemotherapy regimen. On presentation, her visual acuity was counting finger in the right eye and 6/ 6 in the left eye. Both anterior segments were unremarkable. Fundus examination of the right eye showed multiple choroidal masses with the largest in the temporal to fovea. Generally, she was well. Her tumor markers were raised. Urgent Computed Tomography (CT) Scan of thorax, abdomen and pelvis showed multiple distance metastases. She was referred to the gynecology team. She was scheduled for chemotherapy. However, she defaulted the treatment. 3 months after that, her general condition deteriorated. She developed bilateral internal jugular vein thrombosis and massive right pleural effusion. She passed away due to that complication.

**Conclusion:** Choroidal metastasis from primary ovary carcinoma is very rare. Ocular symptoms can be the first presenting features to a life-threatening condition.

## Introduction

Choroidal metastases are considered common intraocular malignancies in adults due to the abundance in vascular supply and hematogenous dissemination from remote major sites. Choroidal metastases account for 88% of cases, followed by iris and ciliary body for metastases to uveal tract and two most common sources of tumor are breast carcinoma followed by lung cancer [**1**,**2**]. However, the spreading of ovarian carcinoma to the uveal tissue, specifically the choroid, is very rare. We reported a case of choroidal mass secondary to mucinous cystadenocarcinoma of ovary in a young woman.

## Method

A case report.

## Result

A 21-year-old woman presented with insidious painless, progressive, central scotoma of the right eye for 5 weeks. The left eye vision was good. She was diagnosed with mucinous cystadenocarcinoma of right ovary 9 years before. She underwent salpingo-oophorectomy and completed 6 cycles of chemotherapy regimen. Afterwards, she was subsequently on yearly gynecological follow up and was disease free for 9 years. Serial imaging of Computed Tomography (CT) scan of the thorax, abdomen, peritoneum, and tumor marker CA 125 were monitored yearly. The latest gynecological follow up was 3 months prior to the ocular presentation. All the investigations results were unremarkable.

**Fig. 1:**
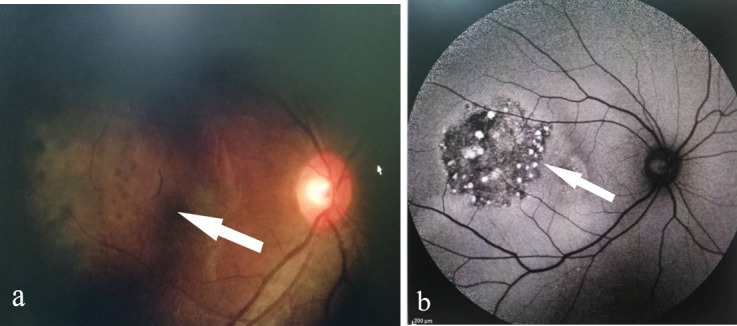
**(a)** Fundus OD; arrow showing large hypopigmented choroidal lesion over fovea extending temporally, corresponding to multiple patchy small hyperfluorescence on hypofluorescence area of temporal fovea **(b)**

**Fig. 2:**
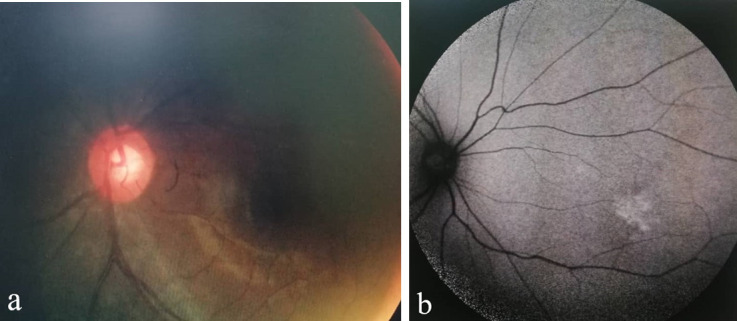
**(a)** Fundus photo and **(b)** Fundus autofluorescence of left eye appeared normal

**Fig. 3:**
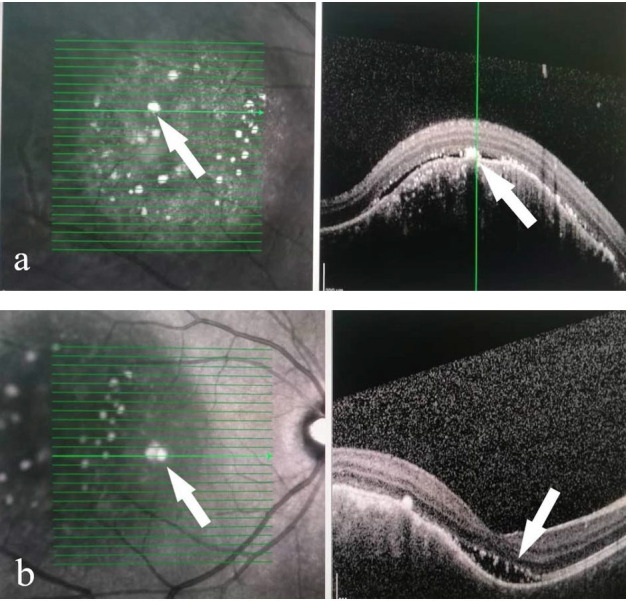
**(a)** OCT macula (lesion area) showing raised choroidal lesion. Multiple hyperreflective spots (arrow) corresponding to the hyperpigmented area on the fundus view located in the subretinal layer with minimal surrounding subretina fluid (hyporeflective layer). **(b)** OCT macula; arrow showing subretinal fluid over the central fovea area

On presentation, her visual acuity was counting finger in the right eye and 6/ 6 in the left eye. Both anterior segments were unremarkable. Fundus examination of the right eye showed multiple choroidal masses with the largest in the temporal to fovea. It was about 5-disc diameters in size, surrounded with multiple hyperpigmented lesions (**Fig. 1 (a), (b)**). Another smaller lesion about 2-disc diameters in size was located nasal to the optic disc. However, there were no vasculitis changes or any retinitis lesions. The left fundus was normal (**Fig. 2 (a), (b)**). Optical Coherence Tomography (OCT) showed elevated choroidal lesion located temporal to fovea with subretinal fluid over the central fovea (**Fig. 3 (a), (b)**).

Systemic examination revealed no lymphadenopathy or any hepatosplenomegaly. Both breast examinations were normal. The latest tumor markers CEA (2.3 gn/ ml) and CA 125 (45 UmL) were elevated. Urgent CT scan of the thorax, abdomen and pelvis revealed the presence of multiple new lesions over the right adnexa, which appeared as a cystic lesion with wall and mural nodule enhancement, measuring 2.0 cm x 2.5 cm. Multiple hypodense nodules were seen at the right hilar region of the lung, liver, and pancreas. Multiple sclerotic bony erosions were also observed over the vertebral body, T10, L2, right iliac wing, left femoral head and left acetabulum. CT scan of the brain was unremarkable.

She was referred to the gynecology and oncology team. She was scheduled for chemotherapy. However, she defaulted the treatment. Three months later she presented to emergency department for shortness of breath, back pain, and jaundice. She developed bilateral internal jugular vein thrombosis and massive right pleural effusion. Her condition deteriorated and she passed away few days later. Permission for autopsy was declined by the family members.

## Discussion

Choroidal metastases are a common intraocular malignancy of adult population [**2**]. The common primary site for tumor sources comes from breast (incidence of 37% to 41%), followed by lung carcinoma (incidence of 2% to 7%) [**3**,**4**]. The case of choroidal metastasis secondary to primary mucinous cystadenocarcinoma of ovary is very rare [**5**]. A review on literature highlighted that only eight cases of metastatic ovarian carcinoma involving the eye have been published, and only one of them was related to mucinous ovarian cystadenocarcinomas [**6**]. 

Choroidal metastasis usually represents hematogenous spread, however, for ovarian carcinoma, a characteristic spread is via intraperitoneal dissemination [**6**]. Our patient had visual symptoms at the initial presentation and subsequently, the CT of the thorax, abdomen and peritoneum revealed intraperitoneal involvement, which included liver and pancreas.

To enlighten our knowledge, the initial symptoms of choroidal metastases are like the ones of other metastases, but the ones from mucinous ovarian cystadenocarcinoma have rarely been reported. Furthermore, the patient was disease free for almost 9 years before she started to look for treatment only for her sight problem. The wound indicated the ocular presentation as an “alarm” for the clinician to think of more other life-threatening metastases from the primary tumor. The blurred vision related to metastasis from ovary carcinoma can be due to choroidal mass causing neurosensory detachment or cancer-related retinopathy, as well as cancer-related optic neuropathy [**7**,**8**].

Apart from blurred vision, other possible ophthalmic symptoms include diplopia, photophobia, ptosis, blepharitis, metamorphopsia, pain, flashes and floaters, mass lesion, uveitis, exophthalmos, secondary glaucoma, and detached retina [**1**]. 

The diagnosis of choroidal metastasis is mainly established by ocular features and associated systemic findings. Ocular examination will reveal a unilateral involvement, commonly, solid, flat, yellow-brown lesion on slit lamp examination. Supplementation of ocular findings with investigations such as Optical Coherence Tomography (OCT), Fundus Autofluorescence (FAF), Fundus Fluorescence Angiography (FFA), as well as fine-needle aspiration biopsy help to support the diagnosis of choroidal metastases [**9**,**10**]. Computed Tomography and Magnetic Resonance Imaging (MRI) are often required for the detection of primary pathology and extension or metastasis. In enhanced depth imaging, OCT of the choroidal lesion will show low internal optical reflectivity with overlying choriocapillaris thinning, irregular, elongated photoreceptor, and presence of subretinal fluid and thickening of retinal pigment epithelium [**11**]. As for our case, we used spectrum domain SD-OCT, which showed similar findings with the previously reported cases of choroidal metastasis, including hyperintense irregular spots of the photoreceptor layer and retinal pigment epithelium, as well as the presence of subretinal fluid [**12**]. Most of the time, FAF imaging revealed hyperautofluorescence in areas of focal pigmentation and subretinal fluid with hypoautofluorescent margins corresponding to the OCT evidence of retinal pigment epithelial (RPE) thickening and subretinal fluid [**13**]. 

Treatment for choroidal metastasis becomes challenging as most of the time the patient with ocular features might as well have a central nervous system or intraperitoneal spread. Thus, treatment options might depend on primary disease status, size and site of choroidal lesion, patient’s symptoms, and general health condition. Systemic treatment always plays the main role in choroidal metastasis regardless of any primary site of tumor as this systemic medication freely diffuses into the choroid via the fenestrated endothelium of choriocapillaris. Many literature data have proven that the lesion of choroidal metastasis regression can be achieved with systemic medications alone [**14**]. Systemic treatment is achieved by either conventional chemotherapies or targeted therapies, which promise more favorable outcomes in terms of reducing side effects of chemotherapy [**14**]. Local therapies for choroidal metastasis should be considered with the input from the multidisciplinary team, involving the patients themselves, oncologist, ophthalmologist, and radiation oncologist. Examples of local therapy options are radiotherapy (external beam, plaque brachytherapy, Gamma knife, and proton beam), intravitreal injection, laser therapy, cryotherapy, or resection [**14**]. 

As for this case, we were able to perform a thorough fundus slit lamp examination, optical coherence tomography (OCT), and photo for documentation, together with fundus autofluorescence. However, in view of the psychosocial issue for the patient, and the acceptance of the disease progression by family members, she chose to abandon subsequent follow up and defaulted the treatment plan. She presented again, 3 months later, with more life-threatening complications that caused her death. 

Generally, survival is poor after the diagnosis of ocular metastasis. Median survival rate after the detection of ocular metastasis is around 5 to 32 months [**10**]. Regardless of primary site, overall prognosis is poor and the 5-year survival estimate of 23% [**10**]. 

## Conclusion

Choroidal metastasis from primary ovary carcinoma is very rare and should be in the list of differential sources of primary tumor in a young woman. Painless unilateral reduced vision in the disease-free patient, which was previously treated as ovarian cancer, can be the first presenting feature to indicate disease recurrence and an indicator for another intraperitoneal metastasis. High index of suspicion may lead to the early detection, subsequently improving the patient’s state and relative acceptance and helping in further planning of management even with limited life expectancy. 

**Conflict of Interest statement**

Authors state no conflict of interest.

**Informed Consent and Human and Animal Rights statement**

Informed consent has been obtained from all individuals included in this study.

**Authorization for the use of human subjects**

Ethical approval: The research related to human use complies with all the relevant national regulations, institutional policies, is in accordance with the tenets of the Helsinki Declaration, and has been approved by the review board of the School of Medical Sciences, Health Campus, Universiti Sains Malaysia.

**Acknowledgements**

None.

**Sources of funding/ Financial support and sponsorship**

None. 

**Disclosures**

None.
